# Effects of Partial-Contact Tool Tilt Angle on Friction Stir Welded AA1050 Aluminum Joint Properties

**DOI:** 10.3390/ma16114091

**Published:** 2023-05-31

**Authors:** Mahmoud E. Abdullah, M. Nafea M. Rohim, M. M. Mohammed, Hamed Aghajani Derazkola

**Affiliations:** 1 Mechanical Department, Faculty of Technology and Education, Beni-Suef University, Beni-Suef 62511, Egypt; mohamed.rahim@techedu.bsu.edu.eg (M.N.M.R.); moustafa.mahmoud@techedu.bsu.edu.eg (M.M.M.); 2 Department of Mechanics, Design and Industrial Management, University of Deusto, Avda Universidades 24, 48007 Bilbao, Spain

**Keywords:** tool tilt angle, friction stir welding, AA 1050, mechanical properties, welding parameters, microstructure

## Abstract

This study aims to investigate the impact of partial-contact tool tilt angle (TTA) on the mechanical and microstructure properties of the AA1050 alloy friction stir weld (FSW). Three levels of partial-contact TTA were tested, 0°, 1.5°, and 3°, compared to previous studies on total-contact TTA. The weldments were evaluated using surface roughness, tensile tests, microhardness, microstructure, and fracture analysis. The results show that in partial-contact conditions, increasing TTA decreases the generated heat in the joint line and increases the possibility of FSW tool wear. This trend was the opposite of joints that were friction stir welded via total-contact TTA. The microstructure of the FSW sample was finer at higher partial-contact TTA, while the possibility of defect formation at the root of the stir zone in higher TTA was more than in lower TTA. The robust sample prepared at 0° TTA had 45% of AA1050 alloy strength. The maximum recorded heat in 0° TTA was 336 °C and the ultimate tensile strength of this sample was 33 MPa. The elongation of the 0° TTA welded sample was 75% base metal, and the average hardness of the stir zone was 25 Hv. The fracture surface analysis of the 0° TTA welded sample consisted of a small dimple, indicating the brittle fracture mode.

## 1. Introduction

The friction stir welding (FSW) process is a solid-state welding technology used in many industries recently for several reasons, such as high productivity, low defects related to melting, and high welding strength [1]. A rotational non-consumable tool penetrates inside the weld seam in the FSW process to join workpieces [2]. The friction between the tool and workpiece increases the weld line temperature and converts the base materials to a pasty form. After that, the FSW tool starts to move forward along the joint line [3]. During the forward movement, the raw material from the tool’s leading edge (LE) extrudes inside the joint area and fills the tool’s trailing edge (TE) [4].

The main FSW process parameters that directly affect the quality of joint material flow and mechanical properties are the FSW tool geometry, rotational velocity, traverse velocity, offset, plunge depth, and tilt angle [5]. One of the most critical FSW parameters that can affect the surface and internal flow is the tool tilt angle (TTA) [6]. TTA is defined as the difference angle between the tool’s normal axis and the normal axis of the workpiece during welding [7]. The TTA can be implemented in two types. The first type is when the whole tool is inside the workpiece (total-contact TTA), and the other type is when part of the tool is inside the workpiece (partial-contact TTA) [8].

In total-contact TTA, the whole shoulder of the tool is in contact with the top surface of the workpiece, and in partial-contact TTA, a part of the tool is not in contact with the workpiece [9]. TTA is a crucial factor that can affect the final properties of the joint. This factor is a leading technical factor during FSW of similar and dissimilar materials [10]. Research shows that TTA significantly impacts the quality of aluminum alloy FSW joints. Due to the wide range of applications of aluminum alloys in various industries, an investigation of the effects of TTA on the final quality of friction stir welded samples is necessary [11]. The TTA was evaluated by researchers in two different categories. The first is related to experimental studies, and the second to simulation studies.

Shah and Badheka [12] studied the effect of total-contact TTA on the tensile strength of AA7075 aluminum alloy welded specimens. It was found that the TTA can be classified as a vital welding parameter due to its ability to control the defects and fracture behavior of welded joints. Acharya et al. [13] evaluated the effect of total-contact TTA on microstructure and microhardness for joining different aluminum series. They reported a difference in the heat-affected and stirred zones with the different tool tilt angles of the welding tool. They also found the microhardness of the weld center slightly higher than the base metal. Birol and Kasman [14] studied the effect of total-contact TTA and tool rotational speed. They found an improvement when increasing the rotational tool speed and reducing the tool tilt angle. Zhai et al. [15] investigated the effect of TTA on material flow and evaluated the defects resulting from the friction stir welding process of AA2219-T6. It was concluded that the best TTA was between 1° and 2°, which made joints defect-free. Gupta et al. [16] implemented 1.5° as a TTA for joining thick AA 7017-T651 aluminum alloy. Several mechanical tests and microstructure analyses were performed to evaluate the efficiency of the welded specimen. It was found that the welding pitch (tool rotational speed/traveling speed) is one of the most significant parameters in controlling the quality of welded specimens at the same TTA.

Acharya et al. [13] investigated the effect of TTA on the mechanical properties and fracture behavior of AA 6092. They found the best TTA in the range 1° to 2°; after that, the strength of joints decreased because of grain growth in the stir zone. It was noted that on increasing the TTA, both the flash formed and the ripple spacing increased. Dialami et al. [17] investigated the effect of total-contact TTA on heat generation and material flow. They found that high heat generation and friction force on the advancing side improved material flow on the retreating side. Zhai et al. [18] investigated the thermo-mechanical modeling of partial-contact TTA during FSW of AA6061 aluminum alloy. During forward movement, they determined an incomplete contact region between the tool shoulder and the workpiece. They measured the incomplete contact region between the shoulder and workpiece and compared it with calculation results.

Among various aluminum alloys, AA1050 aluminum alloy is widely used in various industries such as automotive, aerospace, and railway structures. FSW of AA1050 aluminum can help to produce light structures for various industries. For this reason, investigation of the effects of TTA during FSW of AA1050 alloy seems necessary. Tsarkov et al. [2] investigated the effects of total-contact TTA on the FSW joint of AA1050 alloy. They studied joint configuration with pin-less and regular FSW tools. On the other hand, Barlas [1] investigated the total-contact TTA on FSW of AA1050 aluminum alloy in joint lap configuration.

As mentioned, the difference between total-contact TTA and non-contact TTA is not clearly understood. Due to the available literature, the effects of partial-contact TTA on the AA1050 aluminum alloy have not been considered. In a previous study, the total-contact TTA effects on FSW of AA1050 aluminum alloy were evaluated. These results give base information to compare with other contact conditions of TTA. This study attempts to evaluate the effect of partial-contact TTA on the welding properties and compare the results with total-contact TTA joints. However, it notes a lack of research on the effects of partial-contact TTA on AA1050 aluminum alloy. Therefore, this study aims to fill this gap by evaluating the impact of partial-contact TTA on welding properties and comparing the results with total-contact TTA joints.

## 2. Materials and Methods

**Figure 1:**
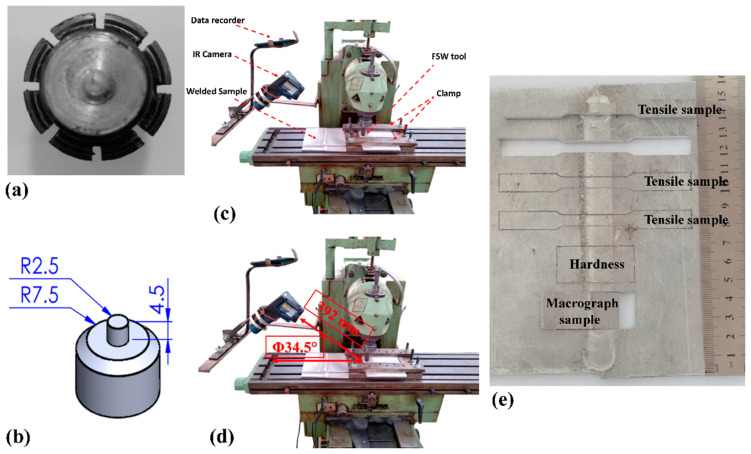
(**a**) Picture of FSW tool and (**b**) Schematic view of FSW tool dimensions. (**c**) Experimental setup of FSW process; (**d**) IR camera angle. (**e**) Mechanical and microstructure test samples.

Commercial pure aluminum 1050 sheets of 5.3 mm thick material were used in this study as weldment. The weldment was provided from a local market, and the chemical composition of the provided aluminum was evaluated in a laboratory. The chemical composition after being tested three times is shown in Table 1. The raw aluminum sheets were cut and made a certain size via a hydraulic shearing machine. The final aluminum pieces were 160 × 100 mm rectangular shapes. Due to the selected parameters, 18 plates were cut and prepared for testing. This number was selected because all FSW conditions were implemented three times and the average values are presented in this study. The welding tool is made from W302 steel with a flat shoulder and cylindrical pin. The tool has a 15 mm shoulder diameter and a 5 mm pin diameter and 4.5 mm length. It has been suggested that the optimum pin length should be approximately 85% of the base metal thickness [19]. Based on previous research, 4.5 mm is the optimum length. A schematic image and a picture of the tool are depicted in Figure 1a and Figure 1b, respectively.

In this study the tool rotational and traverse velocities were selected as 910 rpm and 44 mm/min, respectively. These criteria were selected after undergoing a series of trials. For the assessment of the effects of tool tilt angle, 0°, 1.5°, and 3° tilts were selected. During the experimental procedure, the tilt angle was selected as a variable and other parameters were kept constant. The thermal change during the FSW process was recorded via an infrared thermometer (IR camera). The thermometer was able to record 1000 °C with 0.1 °C resolution and a measuring error of ±1 °C. This type of camera is an easy-to-use comparison with thermocouples due to its applicability in wireless operations and its high responsiveness with respect to up- or down-measured temperature values. During the tests, the IR camera fixed on the machine and focused at a certain point in the advancing side of the joint line. The IR camera was on during the process and recorded the temperature changes of the focused point from the beginning to the end. The thermal changes at the point during the passing of the tool were recorded by the IR camera every 5 s and the data collected in an Excel file. At the end, the recorded numbers were plotted in an Excel file. The total distance between the IR camera and the selected point at the surface of the workpiece was 392 mm with a 34.5° angle. Pictures of the welding setup and the installed IR camera are shown in Figure 1c and 1d, respectively. After the FSW process, each specimen’s average surface roughness (Ra) was measured via the Mitutoyo surface roughness tester at No. of cycles 5.

For evaluation of the microstructure, an optical microscope (OM) was employed. In this study, two types of optical microscopes were used. The first one was an optical stereo microscope (LEICA M165 c) used to evaluate the area of the stir zone, the thermo-mechanical affected zone, and the heat-affected zone in the weld line (micrographic analysis). The second was a light optical microscope (LECO LX 31) connected with a camera Pax-cam for image analysis with software (Pax-it, 1.1).

The hardness of the stir zone was measured according to the Vickers hardness by applying 500 gf for 15 s. The tensile tests of the welded specimen and base metal were performed at room temperature according to the ASTM-E8M standard. The sub-size tensile test specimen dimensions were selected and the test carried out with 2 mm/min crosshead speed. The fracture surface of the tensile tests after testing was evaluated via scanning electron microscope (SEM). Figure 1e presents an image of mechanical and microstructural samples cut from the weld line. After the FSW, the mechanical test samples and metallurgical test samples were cut via a laser cutting machine.

## 3. Results and Discussion

### 3.1. Thermal Cycles

The TTA in FSW significantly impact the material’s surface flow during welding [20]. The tool tilt angle refers to the angle between the rotating tool axis and the normal axis on the workpiece surface [21]. This angle determines the direction and amount of material flow during the FSW process, which affects the weld quality and microstructure [22]. As discussed before, positive TTA, which refers to a tool tilted toward the forward movement, promotes material flow away from the center of the weld, resulting in a wider and shallower weld. On the other hand, a negative tool tilt angle, which refers to a tool tilted in the direction opposite the forward movement, promotes material flow toward the center of the weld, resulting in a narrower and deeper weld. In fact, the contact area between the tool and the workpiece can affect the final product’s quality [23]. The contact area between the tool and the workpiece changes the total heat generation. In this case, the frictional generated heat can change the metallurgical and mechanical properties of the final joint.

**Figure 2:**
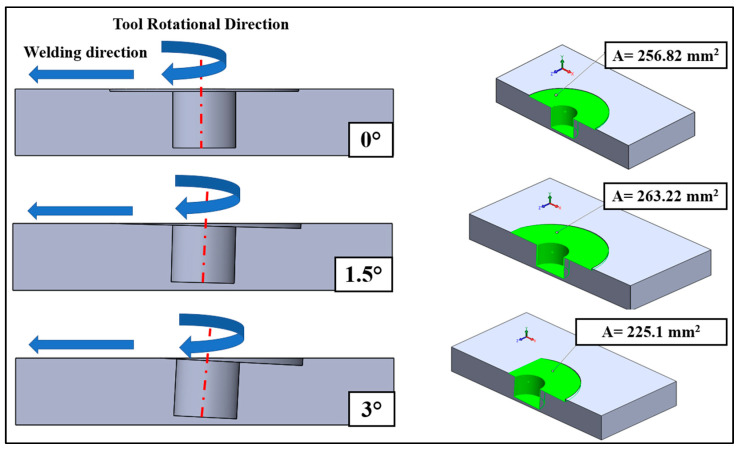
A schematic of the relation between TTA and approximate current contact area at 0.2 mm tool plunging depth.

Geometrical analysis of TTA shows that the highest contact area between the welding tool and the workpiece was at 1.5° TTA (~263.22 mm^2^), while the lowest value of the contact area was at 3° TTA (~225.1 mm^2^) with neglected surface tension or flash formation. This number is calculated according to the contact surface of the tool and aluminum alloy. A schematic view of the calculated contact area is depicted in Figure 2.

**Figure 3:**
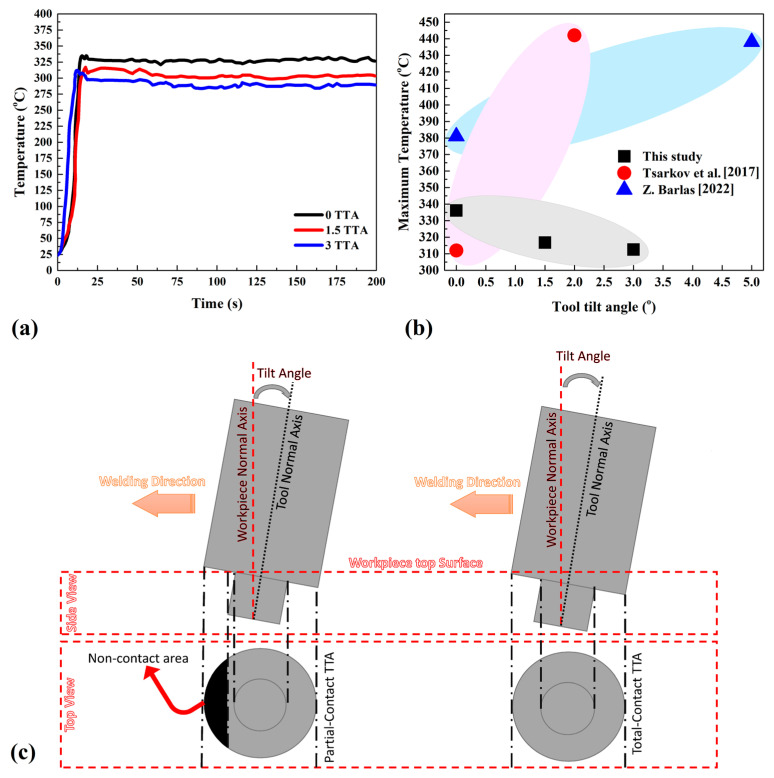
(**a**) Thermal cycles at different TTA. (**b**) Comparison between recorded temperature in this study and two other studies [1,2]; (**c**) schematic view of total-contact TTA and partial-contact TTA.

In this study, the thermal history of all samples was recorded during the welding procedure. By recording thermal history, we intended to find the relation between partial-contact TTA with frictional heat generation during FSW of the AA1050 aluminum alloy. The IR camera was fixed on the advancing side and recorded surface temperature changes from the starting phase of the joining until the ending, and the results are presented in Figure 3a. The results revealed that the temperature rose from room temperature and reached the highest level after that, remaining at the maximum until the end of the welding process. The results show the maximum temperature was recorded in 0° TTA and the minimum temperature was recorded in 3° TTA. In all cases, the recorded temperature peaked and stayed steady after a slight decrease. The steady-state phase in thermal history is related to the traverse length of the tool during the welding procedure. At 0° TTA, the temperature reached 336 °C and steadied between 327 °C and 320 °C along the welding line. The results show that the difference between the maximum and minimum recorded temperature was 16 °C. The temperature difference is related to the contact area between the tool and the workpiece during the traverse movement of the tool. The comparison between the results of this study with previous studies with total-contact TTA is presented in Figure 3b. The recorded maximum temperature in this study was compared with Barlas [1] and Tsarkov et al. [2]. Barlas [1] tested the effects of TTA on the FSW of AA1050 aluminum alloy in joint lap configuration.

As mentioned before, the TTA range in Barlas’ [1] study was 0°, 1.5°, 2.5°, and 5°, while the tool rotational and traverse velocities were 1200 rpm and 30 mm/min, respectively. In this study, only the recorded temperatures in 0° and 5° TTA were reported, which indicates the minimum and maximum temperatures during testing. The test of Tsarkov et al. [2] was almost similar to this study. They used 0°, 1°, and 2° TTA, while the tool rotational and traverse velocities were 900 rpm and 50 mm/min. They reported the maximum and minimum temperatures in their study that were recorded in 0° and 2° TTA. The TTA was in total contact condition and this is helpful in comparing with our study results. The results show that with increasing TTA in partial-contact conditions, the generated heat decreases, while in total-contact conditions with increasing TTA, the total amount of heat increases. As discussed earlier, the difference between partial-contact TTA and total-contact TTA is related to the contact area of the tool and workpieces. A schematic view of total-contact TTA and partial-contact TTA is presented in Figure 3c. As can be seen, the tool and the top of the pin are not in contact with the workpiece during the forward moving of the FSW tool.

**Figure 4:**
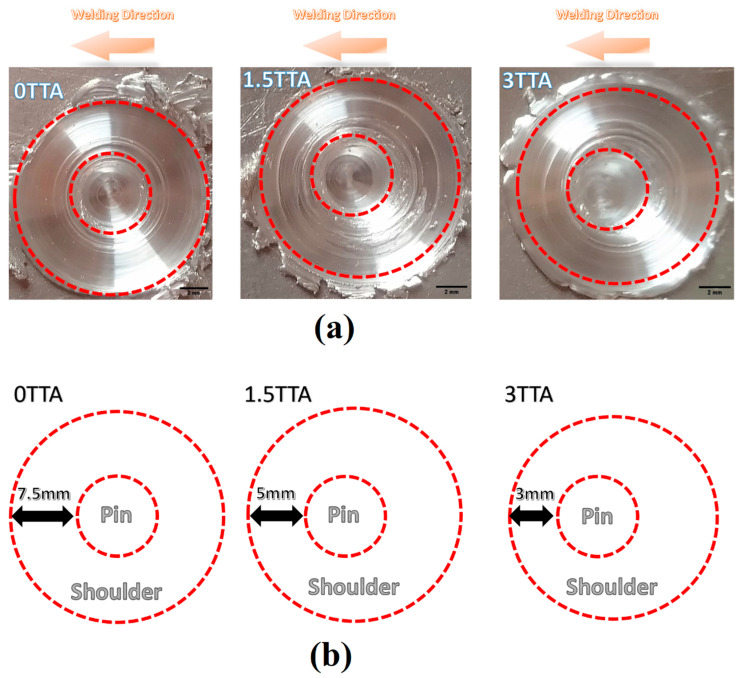
(**a**) The contact surface of the tool and workpiece at 0°, 1.5°, and 3° TTA conditions. (**b**) Distance between leading edge of FSW tool with pin at various TTAs.

The exit holes of 0°, 1.5°, and 3° TTA conditions are presented in Figure 4a. This picture is from the top view of the end of the joint line. The obtained result can help to understand the contact condition between the FSW tool and workpiece at various TTA conditions.

As can be seen from the obtained results, the tool in the sample that welded at 0° was in complete contact with the raw sheets. On increasing the TTA, the contact area at the top of the tool in the leading edge decreased. Due to the partial-contact TTA condition in this study, increasing the TTA decreases the generated frictional heat during welding. The contact length from the shoulder with the leading edge of the FSW tool at various TTAs is presented in Figure 4b. The results show that the distance between the pin and leading edge of the FSW tool at 0°, 1.5°, and 3° TTA is 7.5 mm, 5 mm, and 3 mm, respectively. The results reveal that in partial-contact TTA, the distance between the pin and the leading edge decreases on increasing TTA. In fact, on increasing TTA in the partial-contact condition, the friction surface between the tool and AA1050 aluminum alloy decreases, and generation heat decreases. The frictional heat relates to the contact surface between the tool and the workpiece in the FSW process. Lower surface contact leads to lower heat generation and affects the internal and surface flow.

### 3.2. Surface Flow Analysis

**Figure 5:**
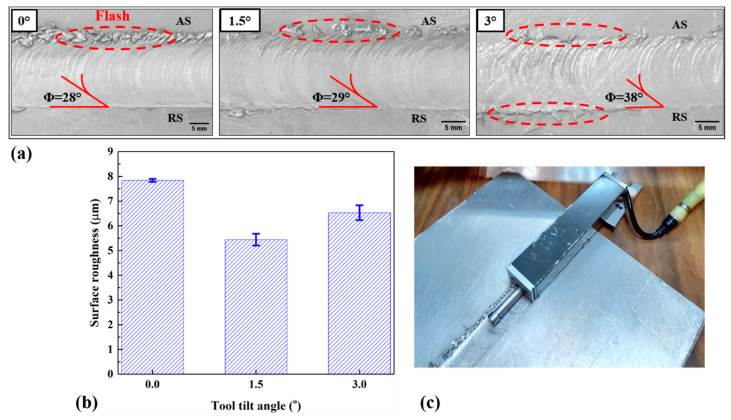
(**a**) Surface flow of joints welded at 0°, 1.5°, and 3° TTA. (**b**) Surface roughness at different TTAs and (**c**) sample of surface roughness measurement.

The surface flow of welded samples was evaluated via visual examination and Mitutoyo surface roughness tester. The visual examination included the effect of TTA on flash forming and surface flow ring. With this test, the effects of partial-contact TTA on the quality of the joint line surface were evaluated. Figure 5a shows the surface top view of the welded specimen at 0°, 1.5°, and 3° TTA. The first look shows that the surface flash formed at all joint surfaces. Surface flash in the FSW refers to a thin layer of material that is extruded from the surface of the welded joint. This material layer forms due to the high temperature and pressure during the FSW process. The surface flash is often visible as a thin, ribbon-like protrusion on the surface of the welded joint, and it can significantly impact the quality and properties of the welded joint. Surface flash can affect the weld quality in several ways, including welded geometry, weld strength, and weld appearance. It was found that at 0° TTA, a flash formed on the advancing side (AS) because the flow of material on the AS was greater than on the retreating side (RS). This flash resulted from mechanical action and heat generation during the welding process. At 1.5° TTA, a flash also formed on the advancing side, but it was not concentrated or not strong compared with 0° TTA. In the case of 3° TTA, a few flashes formed on both sides of the welding line; i.e., they look semi-scattered. This indicated that the effect of TTA at 3° led to a reduction in tool contact area. Based on tilt angle, it was established that the tilt angle has a significant effect with respect to proving the material flow around the welding tool. In partial-contact TTA conditions, the flash formation is related to the contact area between the FSW tool and AA1050 aluminum alloy. In other words, at 0° TTA, the material extruding from the AS is insufficient to make a smooth joint line. In 1.5° TTA, the extrusion of AA1050 from the AS to the RS improved, but in 3° TTA, the big gap between tool and workpiece during forward moving increases the flash surface at both the AS and the RS.

Flow ring is a phenomenon that occurs via surface ripple in the welding area that is in contact with the tool shoulder [24]. The ripple spacing of this study is measured to indicate the visual surface quality of the welded joint as well as the mechanical action in the stir zone. All welded joints have less distinct flow ring deformation. Moreover, it was found that flow rings were discontinuous at 1.5° and 3° due to the TTAs. As can be seen from the obtained results, the angle of the flow rings at 0°, 1.5°, and 3° TTA were 28°, 29°, and 38°, respectively. This shows that on increasing TTA, the surface shear stress on AA1050 aluminum alloy increases, and more shear force is exerted to extrude plasticized metal from the AS to the RS [25]. The results reveal that the flow ring angle from 0° TTA to 1.5° and 3° TTA increased by 3.5% and 35%, respectively. It seems the increase in surface shear stress at higher TTA leads to the formation of surface flash on both the AS and the RS.

The surface roughness was measured via a Mitutoyo surface roughness tester to evaluate the roughness average (Ra) at different TTAs. Figure 5b,c show surface roughness results and a sample of surface roughness measurements. At TTA 0°, the average Ra was 7.84 ± 0.06 µm. In addition, the Ra at 1.5° and 3° TTA was 5.44 ± 0.24 µm and 6.53 ± 0.3 µm, respectively. It was found that the value of Ra at 0° TTA is higher in comparison with other TTAs. This result indicated that more mechanical action occurred at TTA 0°. Tsarkov et al. [2] did not consider surface flow analysis, while Barlas [1] reported that flashes formed in the vicinity of the joint lines welded with low and very high TTAs.

### 3.3. Macrostructure Observation

**Figure 6:**
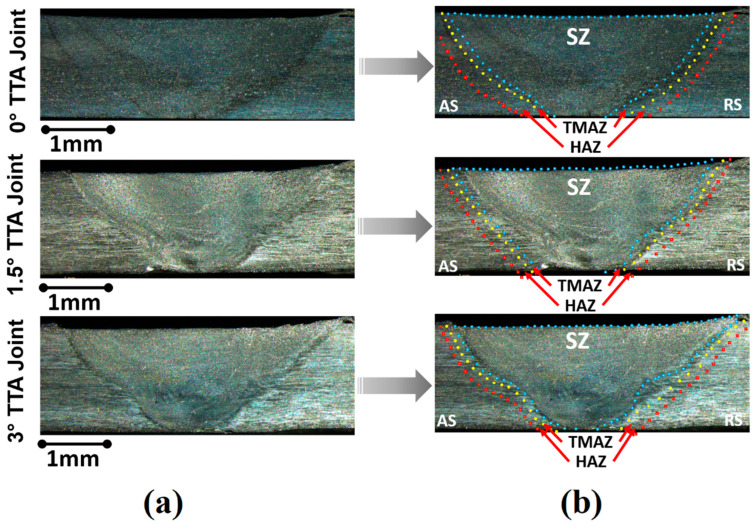
(**a**) Optical macrograph of friction stir joints at various TTAs and (**b**) different areas of joint lines.

Macroscopic images of the cross-sections of the welds at 0°, 1.5°, and 3° are shown in Figure 6a and 6b, respectively. Figure 6a presents cross-section views of FSW joints welded at 0°, 1.5°, and 3° TTA and Figure 6b indicates the different joint areas. Visual inspection revealed that no defects formed in the joint cross-section at a macroscale. Root defects, tunnel voids, or kissing bond defects are common in FSWs of aluminum alloys formed at a macroscale and usually can be detected via visual inspection. As can be seen, in the produced joint, these defects are not detected in cross-sections. As can be seen from the results, the joint line consists of a stir zone (SZ) that is common between the advancing side (AS) and the retreating side (RS). A thermo-mechanical-affected zone (TMAZ) and a heat-affected zone (HAZ) formed at both the AS and the RS. Figure 6b shows the different parts of joint lines. The joint without TTA indicates a symmetrical SZ from the weld center and narrow HAZ and TMAZ areas. The results show the welded area of 0° TTA formed by cup shape. The SZ of joints welded at 1.5° and 3° TTA has similar morphology.

**Figure 7:**
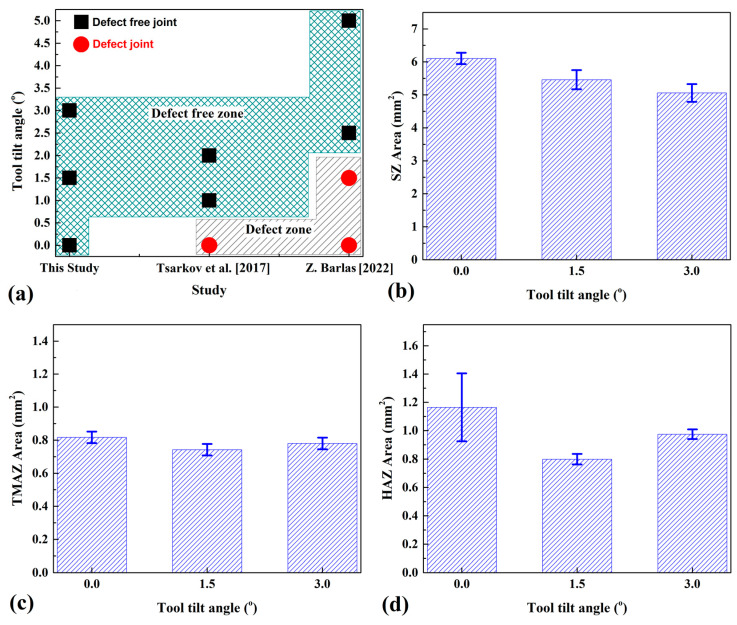
(**a**) Comparison between total-contact and partial-contact TTA joint quality [1,2]. Comparison between total area of (**b**) SZ, (**c**) TMAZ, and (**d**) HAZ at different welded joints.

Figure 7a compares defective and defect-free joints in this study with other studies.

The TTAs used in this research are compared with previous studies, without considering total-contact or partial-contact TTAs. With this comparison, we aimed to understand the effects of TTA quantity and quality on the quality of the joints. The results indicated that defective joints form in total-contact TTA at a low tilt angle, and defect-free joints can be achieved at high TTAs. On the other hand, at least on the macroscale, defect-free joints are achievable in partial-contact TTA joints. This study revealed that partial-contact TTA could achieve a defect-free AA1050 aluminum joint at the macroscale, but in total-contact TTA, sound joints at the macroscale are achievable with a TTA of more than 0°. Figure 7b–d present the thicknesses of HAZ, TMAZ, and SZ of welded samples. The comparison between various joints indicated that the areas of SZ welded at 0°, 1.5°, and 3° TTA were 6.104 mm^2^, 5.46 mm^2^, and 5.059 mm^2^, respectively. The results revealed that the size of the SZ decreased by 17% from 0° to 3° TTA. The TMAZ and HAZ areas were different from the SZ trend. The results show that the TMAZ and HAZ areas decreased from 0° to 1.5° TTA and, after that, increased from 1.5° to 3° TTA. The TMAZ areas of the joints welded at 0°, 1.5°, and 3° TTA were 0.817 mm^2^, 0.742 mm^2^, and 0.78 mm^2^, respectively. On the other hand, the HAZ areas of the joints welded at 0°, 1.5°, and 3° TTA were 1.165 mm^2^, 0.799 mm^2^, and 0.975 mm^2^, respectively.

### 3.4. Internal Flow Analysis

**Figure 8:**
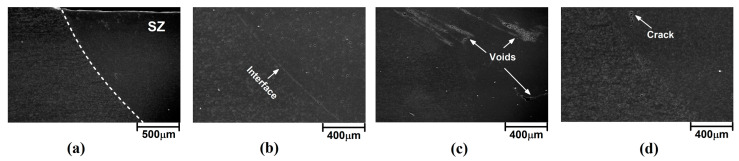
(**a**) Macroscale SEM image of internal flow of friction stir welded sample at 3° TTA. SEM image of SZ interface of friction stir welded sample welded at (**b**) 0° TTA, (**c**) 1.5° TTA, and (**d**) 3° TTA.

The internal flow pattern of welded samples on a microscale gives useful information about the defect formation and voids [26]. For this reason, the internal flow pattern of welded samples was assessed via scanning electron microscope (SEM). A sample SEM image of the welded sample with 3° TTA is presented in Figure 8a. As can be seen, the area of the stir zone and other parts are distinguished clearly. The interfaces of the stir zone and other parts of welded samples with 0°, 1.5°, and 3° TTA are presented in Figure 8b, 8c and 8d, respectively. The depicted SEM image was recorded from the advancing side of the welded sample. The results presented for the advancing side and the retreating side were similar. The results revealed that small voids are formed at the interface of the stir zone and TMAZ for welding at 0° TTA joints. Micro-voids were formed in the stir zone of the joint friction stir welded at 1.5° TTA, and micro-cracks were formed inside the stir zone of the joint friction stir welded at 3° TTA.

**Figure 9:**
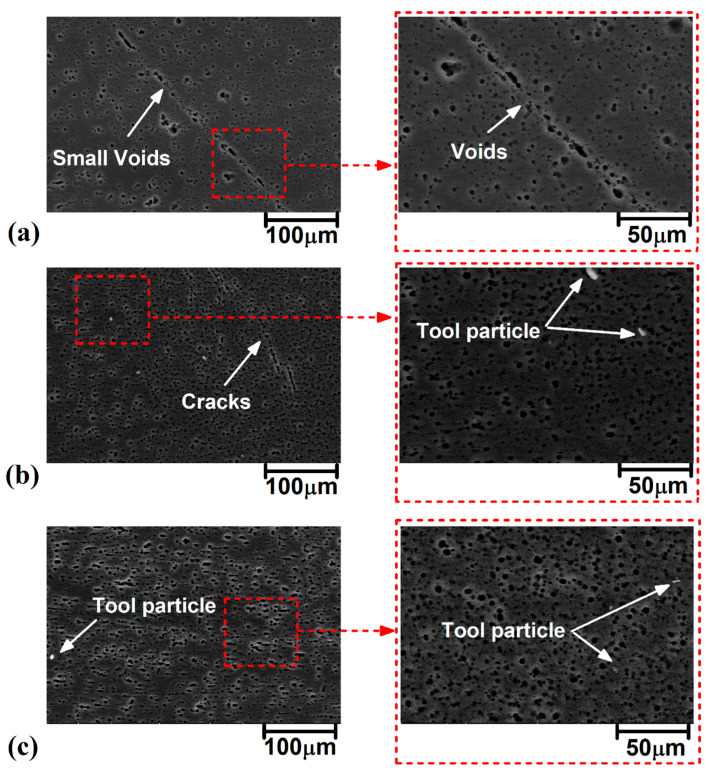
High magnification SEM image of SZ interface of friction stir welded sample welded at (**a**) 0° TTA, (**b**) 1.5° TTA, and (**c**) 3° TTA.

High-magnification images of internal flow of friction stir welded samples welded at 0°, 1.5°, and 3° TTA are presented in Figure 9a–c, respectively. The results show that small voids formed near the stir zone of the sample friction stir welded at 0° TTA. This void seems to result from chemical changes in the AA1050 aluminum alloy after severe plastic deformation with the FSW tool. This trend can be seen in other samples. The results reveal that the size and distribution of these voids decrease at higher TTA. The results show that high non-contact TTA increases the possibility of FSW tool wear. For this reason, shiny particles can be detected, as in Figure 8b,c. On the other hand, the small shiny point can be detected at 1.5° and 3° TTA, which indicates that a small particle of welding tool remained in the joint area.

### 3.5. Microstructure Analysis

**Figure 10:**
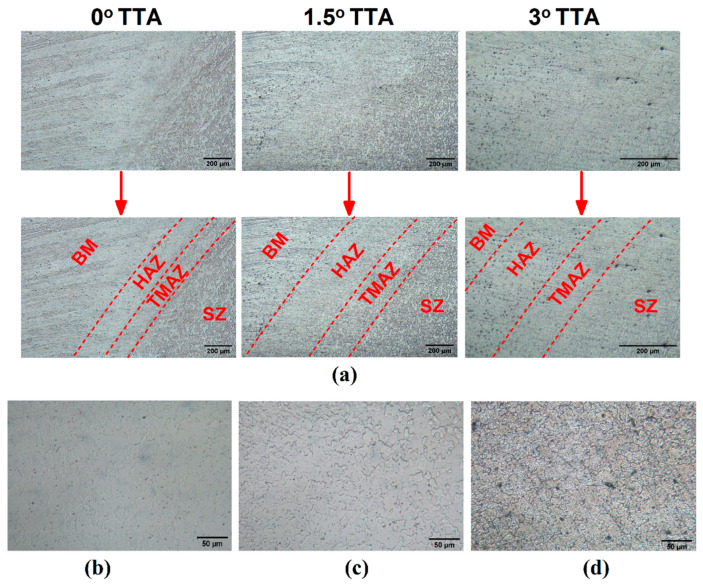
(**a**) Optical image from welding areas of friction stir welded joint at 0°, 1.5°, and 3° TTA. High magnification optical microscope image of SZ microstructure in (**b**) 0°, (**c**) 1.5°, and (**d**) 3° TTA samples.

The microstructure of the weld zone in the FSW process is related to the stirring action of the tool [7]. Severe plastic deformation can change the grain size and microstructure of AA1050 aluminum alloy after FSW. Figure 10a presents a high-magnification optical microscopy image of the various areas of the joint lines. The SZ, TMAZ, and HAZ areas for 0°, 1.5°, and 3° TTA are presented. The microstructures of friction stir welded samples welded at 0°, 1.5°, and 3° TTA are presented in Figure 10b–d, respectively. The presented results are high-magnification images captured via an optical microscope from the centre of the SZ. The average grain size of the SZ was found via image processing software (Version 1.1). As the results show, the average grain sizes of the friction stir welded sample at 0°, 1.5°, and 3° TTA are 51 µm, 58 µm, and 62 µm, respectively. The microstructure of the 0° TTA joint was 17% smaller than the 3° TTA joint. The total contact of the FSW tool with AA1050 aluminum alloy seems to form fine grains in SZ. On increasing TTA, the contact area of the tool shoulder in the leading edge decreased, and the main parts of the tool for stirring action were the pin and back side of the FSW tool in the trailing edge. A lower contact area leads to lower thermo-mechanical action and the formation of bigger grain sizes in the SZ.

### 3.6. Microhardness

**Figure 11:**
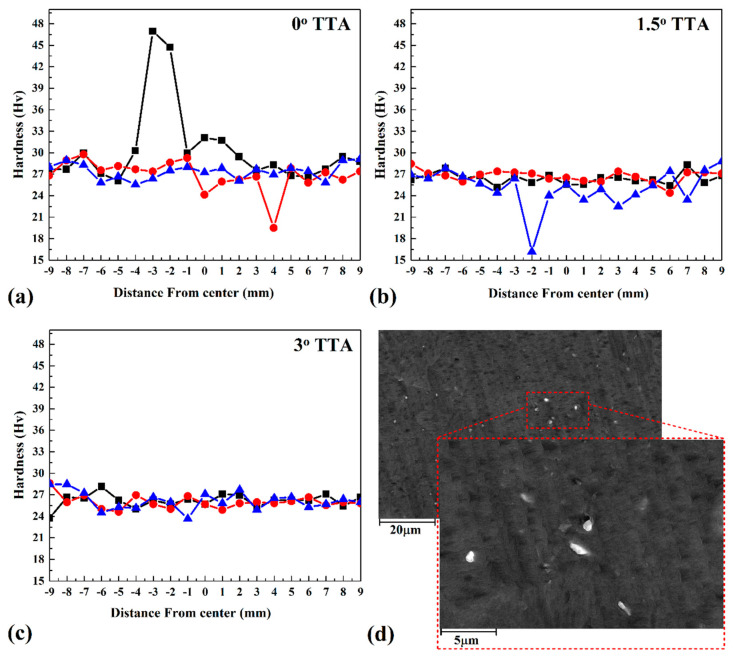
Microhardness profile of specimens friction stir welded at (**a**) 0°, (**b**) 1.5°, and (**c**) 3° TTA. Particle remainder of the FSW tool in the SZ of the 3° TTA joint. (**d**) SEM image of the FSW particles that remained in the SZ.

The hardness of the welding area gives information about the mechanical properties of the final FSW joint [27]. Figure 11a–c indicate the hardness of the welded area that was welded three times. The vertical line from the centre of the SZ was selected for hardness evaluations. The results revealed that the hardness of the HAZ area was slightly lower than other parts in all joints. On the other hand, the SZ is the hardest area in all cases. The results show that the average hardness of the friction stir welded sample with 0° TTA is more than other cases, and the average hardness of 1.5° TTA is lower than other samples. The average hardnesses of the SZ sample welded at 0°, 1.5°, and 3° TTA were 28 Hv, 26 Hv, and 25 Hv. The average hardnesses of the TMAZ of samples friction stir welded at 0°, 1.5°, and 3° TTA were 26 Hv, 24 Hv, and 23 Hv, respectively. On the other hand, the average hardnesses of the HAZ area of friction stir welded samples were 25 Hv, 23 Hv, and 24 Hv at 0°, 1.5°, and 3° TTA, respectively. The hardness difference between 1.5° and 3° TTA is related to the tool particles that remained in the SZ. In partial-contact TTA, the applied force in the FSW tool increases, and the wear rate of the tool is high. In this case, particles remaining in the SZ increase the hardness of the SZ locally. SEM images of tool particles in the SZ are presented in Figure 11d. As can be seen, the shiny particles are remainders of the tool material in the SZ. The used aluminum alloy in this study is categorized as 1XXX aluminum alloy, a low-element aluminum alloy. This means that this aluminum category’s chemical interaction and precipitate formation are very low. On the other hand, in SEM images, the high-weight elements can be seen as shinier than light elements. The tool used was steel, and after FSW, a worn surface can be detected in the tool’s pin. In this case, it can be concluded that the shiny particles in the SZ detected in the SEM image would be small particles of the FSW tool that remained in the SZ. Barlas [1] considered the hardness of the optimum joint in his study. As mentioned earlier, the optimum TTA in Barlas’ [1] study was 2.5° TTA, and the hardnesses of the SZ, HAZ, and AA1050 aluminum alloy were 31.5 Hv, 28.3 Hv, and 40.7 Hv, respectively. The hardness of the SZ in the Barlas [1] study was near this investigation’s result (28 Hv hardness in the stir zone of 3° TTA joint).

### 3.7. Tensile Strength

**Figure 12:**
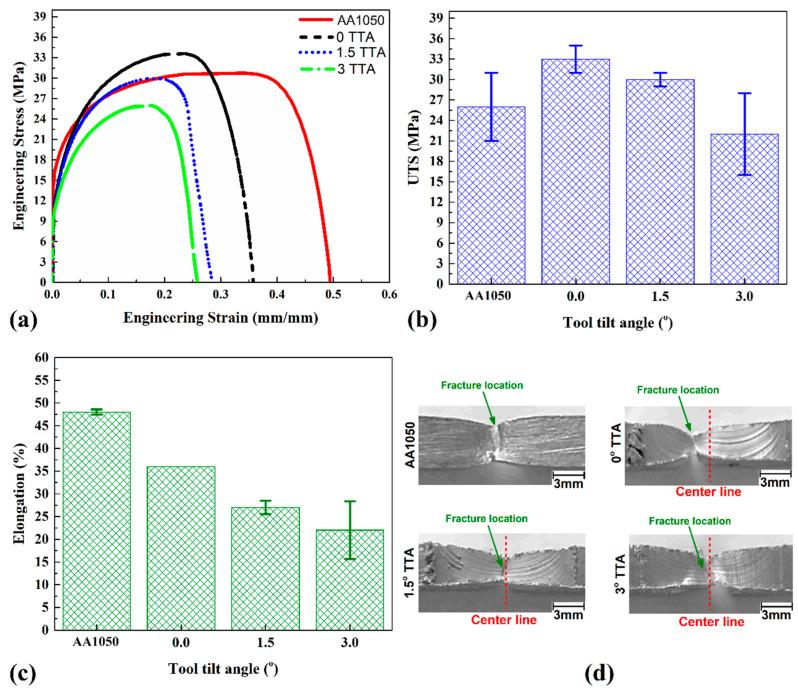
(**a**) Engineering stress–strain curves of base metal and welded specimens, (**b**) UTS and (**c**) elongation of raw metal and welded samples. (**d**) Fracture locations of base metal and welded samples.

Figure 12a shows engineering stress–strain curves of welded specimens at different TTAs and AA1050 aluminum alloys. The obtained results from tests such as ultimate tensile strength (UTS) and elongation percentage (E%) are presented in Figure 12b,c, respectively. As can be seen from the obtained results, the UTS of AA1050 aluminum alloy and the friction stir welded samples welded at 0°, 1.5°, and 3° TTA were 26 MPa, 33 MPa, 30 MPa, and 22 MPa, respectively. The results show that the UTS of friction stir welded samples welded at 0° and 1.5° TTA increased more than base metal, while the UTS of the 3° TTA joint was lower than base metal. It is found that the differences between AA1050 aluminum alloy and friction stir welded samples were +7 MPa, +4 MPa, and −4 MPa for friction stir welded samples at 0°, 1.5°, and 3° TTA, respectively. By dividing the UTS of friction stir welded samples with AA1050 aluminum alloy, it can be found that the joint efficacies of 0°, 1.5°, and 3° TTA samples were ~127%, ~115%, and ~85%, respectively.

**Figure 13:**
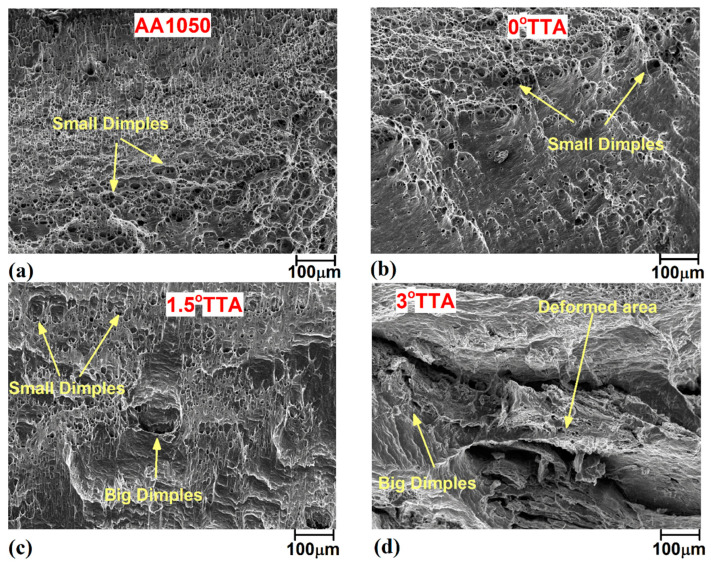
SEM morphology pictures of the fracture surface of tensile tests: (**a**) base metal, (**b**) 0°, (**c**) 1.5°, and (**d**) 3° TTA.

The results indicated that the elongations of AA1050 aluminum alloy and the friction stir welded samples welded at 0°, 1.5°, and 3° TTA were 48%, 36%, 27%, and 22% after the tensile test. By comparing AA1050 aluminum alloy with the friction stir welded samples, it can be concluded that the elongations of welded samples at 0°, 1.5°, and 3° TTA were 75%, 56%, and 50% of raw metal. Figure 12d illustrates the fracture location of AA 1050 alloy and friction stir welded samples after the tensile test. The raw metal fracture place was in the middle of the tensile test sample. The results show that the fracture locations of welded samples at 0°, 1.5°, and 3° TTA were TMAZ, SZ, and SZ areas, respectively. Figure 13a–d show the SEM images from the fracture surface of AA1050 aluminum alloy and welded samples at 0°, 1.5°, and 3° TTA, respectively. Small dimples can be seen in all samples, indicating ductile fracture. On the other hand, a small deformation area can be detected in the FSW sample welded at 0°, 1.5°, and 3° TTA.

## 4. Conclusions

This study aimed to investigate the impact of partial-contact TTA on friction stir welding of AA1050 aluminum alloy. The weldments were examined using several tests such as surface roughness, microstructure analysis, thermal cycles, and tensile tests to achieve the objective of this research. Based on the results of this investigation, the following conclusions are presented:

- The heat generated during the FSW process by increasing the TTA in partial-contact conditions decreases. The maximum heat was recorded at 0° TTA (336 °C) and the minimum heat was recorded at 3° TTA (320 °C). The generated heat decreased due to the smaller contact area between the tool and the AA1050 alloy at the higher partial-contact TTA.
- Increasing TTA in partial-contact conditions decreases the size of the SZ, TMAZ, and HAZ areas. The formed joints were defect-free at a macroscale, while internal defects beneath of pin increase at higher TTAs in partial-contact conditions. Microscale voids and small interface gaps were detected at 3° TTA, while root void defects were not formed at 0° TTA.
- The highest strength joint was produced at 0° TTA, and the weakest joint was produced at 3° TTA. The ultimate tensile strength and average hardness of the joint friction stir welded at 0° TTA were 33 MPa and 26 Hv, respectively. The joint efficiencies of 0°, 1.5°, and 3° TTA samples were ~127%, ~115%, and ~85%, respectively.

## Figures and Tables

**Table 1:** Average chemical composition of pure aluminum 1050 (wt.%).

Element	Fe	Cu	Zn	Ni	Ca	Ga	Pb	B	Al
Wt.%	0.335	0.016	0.007	0.007	0.007	0.007	0.005	0.003	Balance
StdDev.	0.0000000	0.0005774	0.0017321	0.0005774	0.001	0.0005774	0.000	0.0005774	

## Data Availability

Not applicable.
